# Synthesis and Evaluation of 3-Halobenzo[*b*]thiophenes as Potential Antibacterial and Antifungal Agents

**DOI:** 10.3390/ph15010039

**Published:** 2021-12-28

**Authors:** Prerna J. Masih, Tanay Kesharwani, Elivet Rodriguez, Mia A. Vertudez, Mina L. Motakhaveri, Terelan K. Le, Minh Kieu T. Tran, Matthew R. Cloyd, Cory T. Kornman, Aimee M. Phillips

**Affiliations:** 1 Department of Biology, University of West Florida, 11000 University Pkwy, Pensacola, FL 32514, USA; elivet.rdz@gmail.com (E.R.); alaynamv1@gmail.com (M.A.V.); mmotakhaveri@umc.edu (M.L.M.); tkl6@students.uwf.edu (T.K.L.); mtt15@students.uwf.edu (M.K.T.T.); legendaryraycharles@gmail.com (A.M.P.); 2 Department of Chemistry, University of West Florida, 11000 University Pkwy, Pensacola, FL 32514, USA; tkesharwani@uwf.edu (T.K.); mrc53@students.uwf.edu (M.R.C.); ctkornman@gmail.com (C.T.K.)

**Keywords:** antimicrobial, antibacterial, antifungal, benzo[*b*]thiophene, ADME, time-kill

## Abstract

The global health concern of antimicrobial resistance has harnessed research interest to find new classes of antibiotics to combat disease-causing pathogens. In our studies, 3-halobenzo[*b*]thiophene derivatives were synthesized and tested for their antimicrobial activities using the broth microdilution susceptibility method. The 3-halo substituted benzo[*b*]thiophenes were synthesized starting from 2-alkynyl thioanisoles using a convenient electrophilic cyclization methodology that utilizes sodium halides as the source of electrophilic halogens when reacted along with copper(II) sulfate. This environmentally benign methodology is facile, uses ethanol as the solvent, and results in 3-halo substituted benzo[*b*]thiophene structures in very high yields. The cyclohexanol-substituted 3-chloro and 3-bromobenzo[*b*]thiophenes resulted in a low MIC of 16 µg/mL against Gram-positive bacteria and yeast. Additionally, in silico absorption, distribution, metabolism, and excretion (ADME) properties of the compounds were determined. The compounds with the lowest MIC values showed excellent drug-like properties with no violations to Lipinski, Veber, and Muegge filters. The time-kill curve was obtained for cyclohexanol-substituted 3-chlorobenzo[*b*]thiophenes against *Staphylococcus aureus*, which showed fast bactericidal activity at MIC.

## 1. Introduction

There has been an increasing concern over the rapidly spreading resistance to existing antibiotics and antifungal drugs [1,2]. The antimicrobial resistance has been driven by antimicrobial exposure via underuse, overuse, and misuse in health care (human and veterinary medicine), agriculture, aquaculture, and the environment [3]. Antibiotic resistance threats report at least 18 antibiotic-resistant strains of bacteria and fungi. According to the Centers for Disease Control (CDC), 2.8 million people in the United States are infected with antibiotic-resistant bacteria or fungi each year, and over 35,000 of them die. The estimated national cost to treat infections in the USA alone is more than USD 4.6 billion annually [4]. The resistant microorganisms rapidly develop to evade antimicrobial effects by a wide range of complex biochemical and physiological mechanisms [5]. Unfortunately, there has not been a new class of antibiotics approved since the discovery of daptomycin in 1986 [6]. Therefore, we must continue to find new classes of antibiotics to keep up with the ever-changing evolution of pathogens.

Due to its diverse biological activities and drug-like properties, benzo[*b*]thiophene could be an interesting and potential pharmacophore to explore. These core structures are found in naturally occurring organic molecules and exist as a component of several drug molecules, such as raloxifene, zileuton, and sertaconazole. Benzo[*b*]thiophenes exhibit a wide range of biological activities including antimicrobial [7], antifungal [8,9], anticancer [10], antidepressant [11,12], anti-inflammatory [13,14], antioxidant [15], antitubercular [16,17], and anticonvulsant [18]. In addition, benzo[*b*]thiophene derivatives act as histamine H3 antagonists [19], fatty acid amide hydrolase (FAAH) inhibitors [20], and rho kinase inhibitors [21]. Due to its aforementioned biological properties, benzo[*b*]thiophenes has attracted significant attention from synthetic chemists around the globe [22]. Some of the recent efforts in the synthesis of benzo[*b*]thiophene include one-step synthesis of benzo[b]thiophenes by aryne reaction with alkynyl sulfides reported by Yoshida and coworkers [23], the photocatalytic radical annulation process reported by König and coworkers [24], metal-free iodine-catalyzed cascade reactions of alkynes with thiophenols reported by Wang and co-workers [25], and iodine-promoted photocyclization of 4,5-diaryl-substituted thiophenes reported by Fisyuk and coworkers [26].

**Figure 1:**
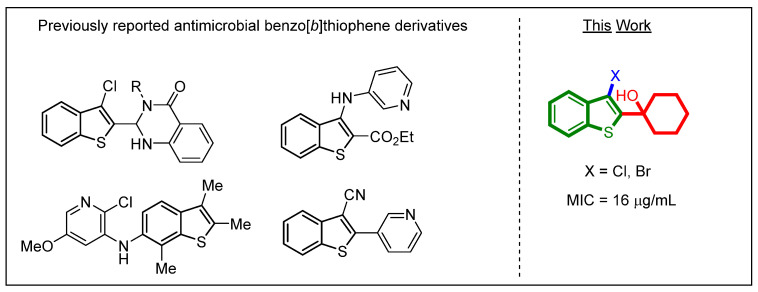
Some previously known benzo[*b*]thiophene derivatives exhibiting antimicrobial activities.

In the past two decades, several studies have explored the antimicrobial activities of benzo[*b*]thiophene derivatives, which more commonly contained other complex pharmacophores, such as quinazolines, coumarins, pyrimidines, carbamates, ureas, semicarbazides, and pyrazoles [7,8,9,27,28,29,30,31,32]. In the literature, the anti-microbial activity of benzo[*b*]thiophene derivatives appeared to be more dependent on substitution at the heterocyclic thiophene ring rather than at the aromatic moiety [18]. The correct placement of the substituents at the third position of the benzo[*b*]thiophene ring is the key to harnessing the desired antimicrobial activity (Figure 1). In the past, researchers have demonstrated that amine [8], amide [31,33], methyl [29,34], ether, and nitrile [35] functionalities enhance the desired antimicrobial activity of the benzo[*b*]thiophene rings. In addition, there have been several reports of the improved antimicrobial activity with the presence of chlorine in the third position [32,36,37]. However, there have been no systematic studies on the effect of other halogens at the third position. Halogen-containing carbo- and heterocycles comprise approximately 40% of drugs that are currently undergoing clinical trials or have been approved as drugs [38,39]. The halogen atoms, particularly chlorine and fluorine, could play important roles in significantly improving the drug-target binding affinity and absorption, distribution, metabolism, and excretion (ADME) properties of a molecule [40,41]. 

In the literature, the targets for benzo[*b*]thiophenes exhibiting antimicrobial properties have not been explored. There is only one study by Liger and coworkers that showed C2-arylated benzo[*b*]thiophene derivatives as a potent NorA pump inhibitor [42]. NorA is an efflux pump resulting in resistance to fluoroquinolones (e.g., ciprofloxacin), quaternary ammonium compounds, biocides, and dyes [43,44]. However, more research is needed to realize the full potential of benzo[*b*]thiophene and its derivatives and to better understand the unique biological properties of this interesting heterocycle.

Therefore, we decided to conduct a systematic structure–activity relationship (SAR) study of various 3-halo substituted (halo = Cl, Br and I) benzo[*b*]thiophene molecules for their antibacterial and antifungal activities to better understand the regiochemical effect of the halogen moiety. Unfortunately, we did not include fluoro-substituted benzo[*b*]thiophenes because the synthesis of 3-fluoro analogues of benzo[*b*]thiophenes has proven to be challenging, and our electrophilic cyclization methodology did not result in the desired fluoro-substituted product. We hereby report the synthesis of novel and simpler halogenated benzo[*b*]thiophene derivatives and the evaluation of their antimicrobial activity and in silico ADME properties. The time-kill kinetics of the selected compound with the lowest minimum inhibitory concentration (MIC) was further investigated.

## 2. Results and Discussion

### 2.1. Synthesis of Benzo[b]thiophene Derivatives

The desired 3-halo substituted benzo[*b*]thiophene derivatives were synthesized using electrophilic cyclization reactions. Electrophilic cyclization of alkynes is a reaction that involves the activation of a C-C triple bond via halogen, boron, sulfur, and selenium electrophiles to undergo cyclization with an internally tethered C, O, N, P, S, or Se nucleophile. Electrophilic cyclization reactions have gained much attention in recent years due to their simplicity in generating various halogenated heterocycles with ease [45]. Larock and others have reported a simple two-step synthesis of 3-iodo- and 3-bromosubstituted benzo[*b*]thiophenes starting from readily available 2-iodothioanisole [46,47]. The first step is the synthesis of 2-alkynylthioanisole via a Sonogashira coupling reaction involving terminal alkyne and 2-iodothioanisoles. The second step consists of the cyclization reaction using I_2_, ICl, Br_2,_ or N-bromo succinimide (NBS) electrophiles. Recently, Kesharwani and co-workers demonstrated that the seemingly difficult chlorocyclization of 2-alkynylthioanisole could easily be achieved using table salt as the source of electrophilic chlorine in the presence of copper sulfate in ethanol. This environmentally benign method has also been demonstrated to work for bromo- and iodocyclization by changing sodium chloride to sodium bromide and sodium iodide, respectively [48,49,50].

**Scheme 1:**
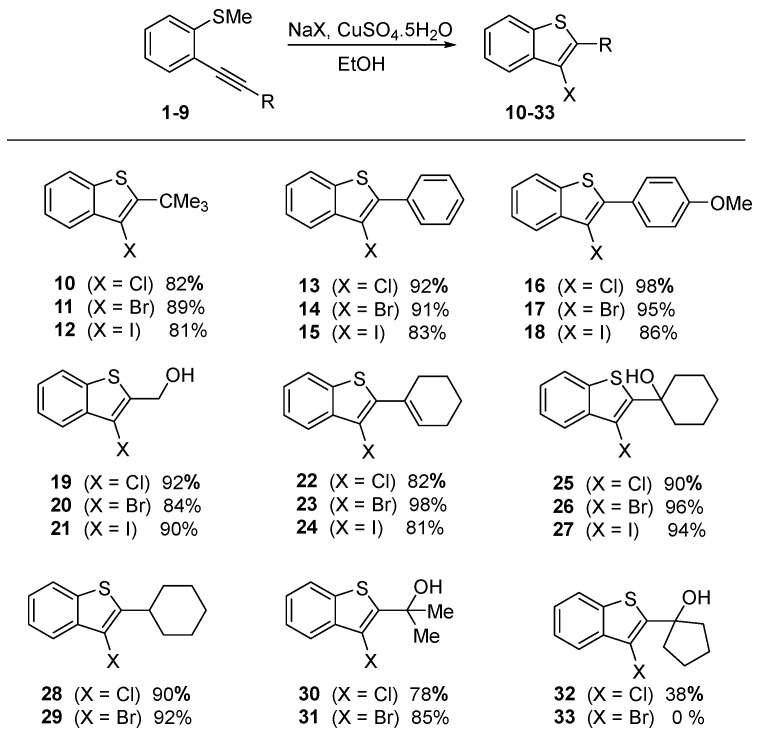
Synthesis of 3-halobenzo[*b*]thiophene derivatives via electrophilic cyclization.

We employed the green halocyclization reaction above to synthesize a series of 3-halo substituted benzo[*b*]thiophenes as depicted in Scheme 1. The synthesis of benzo[*b*]thiophenes **10**–**27** has already been reported earlier [48,49,50]. However, the synthesis of 2-cyclohexyl, 2-(1,1-dimethylmethanol), and 2-cyclopentanol substituted 3-chloro and 3-bromobenzo[*b*]thiophenes derivatives **28**–**32** have not been reported in the literature, and we report their first synthesis herein. The cyclization reaction of cyclohexyl substituted alkynyl thioanisole **7** with sodium chloride and sodium bromide worked with ease to give the corresponding 3-chloro and 3-bromo benzo[*b*]thiophene derivatives **28** and **29** in 90% and 92% yields, respectively. The cyclization reaction of substituted propargyl alcohol **8** resulted in the formation of 2-(3-chlorobenzo[*b*]thiophen-2-yl)propan-2-ol (**30**), and its bromo analogue **31** in excellent yields of 77% and 85%, respectively. The chlorocyclization of alkyne **9** resulted in a lower yield of 35% of the desired benzo[*b*]thiophene **32**. However, our numerous synthetic efforts to cyclize **9** with Br electrophile failed, and the desired bromocyclized product **33** could not be obtained under various conditions. In addition to our green bromocyclization reaction condition, we employed other bromocyclization conditions involving electrophiles such as Br_2_ and NBS. The final product, **33**, seems to be very unstable; the alcohol moiety quickly dehydrates into the corresponding alkene. All of the synthesized compounds (**10**–**33**) were analyzed using ^1^H NMR, ^13^C NMR, and high-resolution mass spectrometry (HRMS). The details of analysis of the newly reported molecules are provided in the Supplementary Materials. 

### 2.2. Minimum Inhibitory Concentration Determination

The benzo[*b*]thiophene derivatives substituted with three halogens, namely chlorine, bromine, and iodine, at the third position were screened to study the effect of halogens on antimicrobial activity. The antimicrobial activity was tested using the broth microdilution method [51,52] to determine the minimum inhibitory concentration (MIC) against three Gram-positive bacterial strains (*B. cereus*, *S. aureus*, *E*three Gram-negative bacterial strains (*E. coli*, *K pneumoniae*, *P. aeruginosa*), and one fungal strain (*C. albicans*) (Table 1).

The 2-*tert*-butyl, phenyl, and 4-methoxyphenyl substituted benzo[*b*]thiophenes **10**–**18** resulted in no significant inhibitory activity against either the bacteria or the fungi. The benzo[*b*]thiophene **19**, with methyl alcohol at the second position and chlorine substitution at the third position, showed better antimicrobial activity against the Gram-positive bacterium, *B. cereus*, and fungus, *C. albicans*, with MIC of 128 µg/mL. Compound **19** also showed moderate inhibitory activity with MIC of 256 µg/mL against the other Gram-positive bacteria, namely *S. aureus* and *E. faecalis*. These data suggested that the alcohol moiety enhanced the activity of benzo[*b*]thiophene derivatives. It should be noted that cyclohexene substituted benzo[*b*]thiophenes **22** and **23** exhibited some inhibitory activity against all the Gram-positive bacteria and the fungus tested. It is known that alkene moieties can undergo hydration under biological conditions, thus further suggesting that the alcohol group could play a significant role in the inhibitory activity of these benzo[*b*]thiophene derivatives. Based on the encouraging activity of methyl alcohol and cyclohexene substituted benzo[*b*]thiophenes, we decided to screen 2-(1-cyclohexanol)-3-halobenzo[*b*]thiophene derivatives **25**–**27**. The chloro- and bromo-containing cyclohexanol compounds, **25** and **26**, had a low MIC of 16 µg/mL against *B. cereus*, *S. aureus*, *E. faecalis*, and *C. albicans*. It was also concluded that out of the three halogens employed, only the bromo- and the chloro-substituted benzo[*b*]thiophenes were active, whereas the iodo substitution did not demonstrate any inhibitory activity up to MIC > 512 µg/mL. We decided not to include the iodo-substituted derivatives further in compounds **28**–**33**, as the previously iodo-substituted compound (**27**) did not show any significant antimicrobial activity compared to bromo-(**26**) and chloro-substituted (**25**) derivatives. To evaluate the significance of the alcohol group, we chose to synthesize and evaluate cyclohexane substituted benzo[*b*]thiophene derivatives **28**–**29**, which showed insignificant or no activity against bacteria (MIC > 512 µg/mL) except for 2-cyclohexyl-3-chlorobenzo[*b*]thiophene (**28**), which resulted in a high MIC of 512 µg/mL against *C. albicans*. This result showed that alcohol contributed significantly to the inhibitory activity of 3-halo substituted benzo[*b*]thiophene compounds, **25** and **26**, against *B. cereus*, *S. aureus*, *E. faecalis*, and *C. albicans*. Finally, we decided to change the cyclohexanol group to cyclopentanol and 2-hydroxypropan-2-yl groups to evaluate further whether the cyclohexanol group is required for high inhibition of bacteria and fungi. The 2-(hydroxypropan-2-yl)-3-chlorobenzo[*b*]thiophene **30** and its bromo analogue **31** resulted in a higher MIC value when compared with corresponding cyclohexanol derivatives **25** and **26**. The MIC value for 2-hydroxypropan-2-yl was 64 µg/mL, whereas the MIC value for cyclopentanol was 128 µg/mL against *S. aureus*, *E. faecalis*, and *C. albicans*.

With all of the above-mentioned inhibitory studies, we concluded that the most active compound had a chloro or bromo halogen group at the third position, and the MIC value was non-different between chloro **25** and bromo derivative **26**. However, chloro-substituted methyl alcohol **19** was most active and 2-(hydroxypropan-2-yl)-3-chlorobenzo[*b*]thiophene **30** was only slightly more active than its bromo analogue **31** in *E. faecalis* and *B. cereus*. In contrast, the iodine-containing molecules did not show any antimicrobial activity until the highest concentration tested. It was also determined that the hydroxymethyl group at the second position of the benzo[*b*]thiophene seems to be important for the inhibitory activity against the Gram-positive bacteria and *C. albicans*. The cyclohexanol structure worked the best, but cyclopentanol and 2-hydroxypropan-2-yl groups were not as effective. It should also be noted that none of the tested compounds showed any activity against Gram-negative bacteria, even at the highest concentration. Gram-negative bacteria have an additional outer membrane structure with the outer-lipid leaflet containing lipopolysaccharides [53]. This makes Gram-negative bacteria more resistant to antimicrobial agents that are unable to cross this additional layer [54] as shown by several studies [55,56].

### 2.3. Time-Kill Kinetics

**Figure 2:**
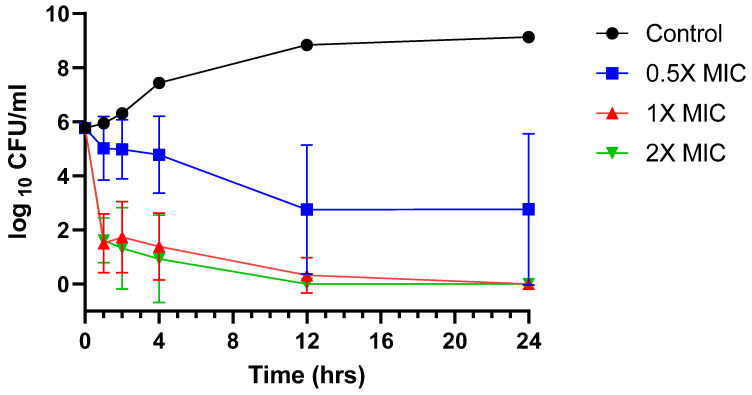
Time-kill curve for compound **25** against *S. aureus*. The data in the graph are depicted as mean ± S.D (*n* = 3, 4).

The cyclohexanol- and halogen- (bromine and chlorine) containing benzo[*b*]thiophenes, **25** and **26**, exhibited the lowest MIC value. However, the microdilution assay provides an end-point result that does not give enough information about the antimicrobial kinetics or whether the tested compound is bactericidal (killing an organism) or bacteriostatic (stalling the growth of an organism). Therefore, we decided to further evaluate the dose and time-dependent killing kinetics of the chloro-substituted cyclohexanol benzo[*b*]thiophene derivative **25** against *S. aureus* at 0.5× (8 µg/mL), 1× (16 µg/mL), and 2× (32 µg/mL) MIC using the time-kill assay [57]. The time-kill assay permits determining the rate of change in the number of viable bacteria compared to the initial starting inoculum at different concentrations of the compound of interest. A time-kill curve for compound **25** against *S. aureus* was plotted as log_10_ CFU/mL versus time (Figure 2). The cutoff point for determining the minimum bactericidal concentration (MBC) was ≥ 3 log_10_ CFU/mL reduction from the starting bacterial density [58].

The time-kill kinetics of compound **25** displayed rapid bactericidal activity towards *S. aureus* at both MIC and twice the MIC concentration within 1 hour of exposure, which resulted in >3log_10_ reduction in viable cell count relative to the initial inoculum (Figure 2). However, 0.5× MIC concentration decreased the viable cells but did show ≤ 3log_10_ reduction. The time-kill assay for compound **25** was consistent with bactericidal characteristics against *S. aureus*. Thus, compound **25** was determined to be a potent bactericidal agent against *S. aureus*. 

### 2.4. In Silico ADME Properties

We analyzed the *in silico* physicochemical and pharmacokinetic properties of the benzo[*b*]thiophene derivatives with lower MIC values (25, 26, 30, 31, and 32) using the freely available Swiss ADME tool). This software gives access to a pool of fast yet modest predictive models that utilize simple molecular and physicochemical descriptors, such as molecular weight (MW), the count of specific types of bonds (the numbers of heavy atoms, aromatic heavy atoms, rotatable bonds, hydrogen-bond acceptors, hydrogen-bond donors), topological polar surface area (TPSA), and several others, which are vital determinants to predict good drug/lead-like molecules [59]. The key calculated/predicted values of common physicochemical parameters of the benzo[*b*]thiophene derivatives are shown in Table 2.

The physicochemical parameters (shown in Table 2) were used to predict the drug-likeness of a molecule. The Lipinski rule-of-five is the most widely used rule-based filter of drug-likeness, which filters the molecules on a range of parameters, namely, molecular weight (MW) < 500 g/mol, hydrogen bond donors (HBDs) < 5, hydrogen bond acceptors (HBAs) < 10, and a logarithm of the octanol/water partition coefficient < 5 or (MlogP < 4.15) [60]. In addition, the number of rotatable bonds (nRotB) of ≤ 10 and a topological polar surface area (TPSA) of ≤ 140 Å^2^ have been included in the Veber filter [61]. Swiss ADME software also includes Ghose (Amgen) [62], Egan (Pharmacia) [63], and Muegge (Bayer) [64] filters for drug-likeness predictions. Table 3 shows the results of the drug-likeness predictions based on all five filters. Interestingly, all analyzed compounds showed excellent drug-likeness with all five filters, with no violation of any of the physicochemical parameters. The molecules were also analyzed to identify potentially problematic or promiscuous fragments that could be putatively unstable, reactive, toxic, or prone to interfere with biological assays. Two complementary pattern recognition methods were implemented, namely PAINS (for pan assay interference structures) [65] and Brenk alerts [66]. None of the compounds analyzed showed any PAINS or Brenk alerts (Table 3).

**Figure 3:**
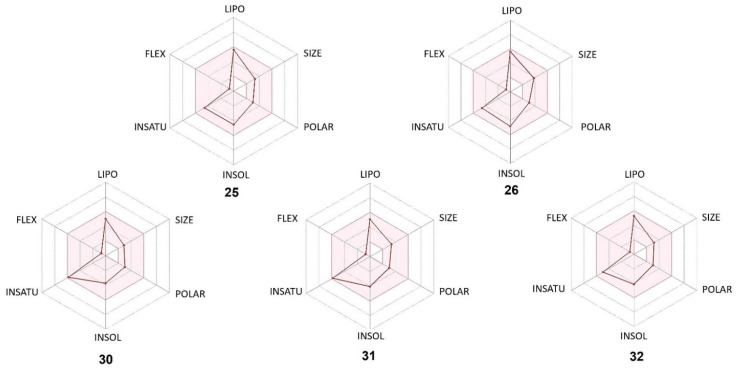
Bioavailability radar plot generated using SWISS ADME is based on lipophilicity (LIPO), size, polarity (POLAR), solubility (INSOLU), saturation (INSAU), and flexibility (FLEX). The analyses of benzo[b]thiophene derivative compounds **25**, **26**, **30**, **31**, and **32** are shown.

In addition, the bioavailability radar plot of the compounds is shown in Figure 3. The pink area of the radar shows an optimum range of six physicochemical properties acceptable for drug-likeness. All of the analyzed compounds, **25**, **26**, **30**, **31**, and **32**, were entirely within the pink area of the radar plot and thus considered to be drug-like.

Further, the pharmacokinetic properties predicted for the benzo[*b*]thiophene derivatives are included in Table 4, namely log of skin permeability (log Kp), blood–brain barrier (BBB) penetration, and gastrointestinal (GI) absorption. All of the compounds analyzed were predicted to have high GI absorption and BBB penetration; thus, these compounds could find applications in the treatment of brain-related infections. 

Additionally, the molecules were analyzed for their interactions with pharmaceutically important proteins, including permeability glycoprotein (P-gp) and Cytochrome P450 (CYP). P-gp primarily functions as an active efflux transporter and is widely distributed in the small intestine, blood–brain barrier capillaries, and several critical organs such as the kidney and liver [67]. It is associated with the efflux of xenobiotics from the brain and multi-drug resistance in cancer cells [68]. The compounds with the lowest MIC values, **25** and **26**, were substrates for P-gp, whereas hydroxypropan-2-yl derivatives **30** and **31** were not the substrates for P-gp. Thus, a substrate of P-gp could be rendered less effective through efflux [69].

Cytochrome P450 (CYP450) is a superfamily of enzymes that plays a crucial role in the metabolism of drugs, steroids, fat-soluble vitamins, carcinogens, pesticides, and many other chemicals [70]. More than 50 isoforms of CYP enzymes exist, with 1A2, 2C9, 2C19, 2D6, and 3A4 isoforms accounting for over 90% of oxidative metabolic processes [71]. During drug development, studying the inhibitory activity of proposed derivatives against certain CYP isoforms is helpful to determine whether the molecules would be efficiently metabolized and cleared. Table 4 shows the results of the inhibitory prediction for five CYP isoforms. None of the proposed compounds inhibited CYP3A4, whereas just five derivatives were found to inhibit CYP2D6. Both CYP1A2 and CYP2C19 were found to be inhibited by all of the benzo[*b*]thiophenes derivatives. In addition, all compounds except **30** and **31** were found to inhibit CYP2C9. Based on the protein interaction with P-gp and cytochrome P450 isoforms, hydroxypropan-2-yl derivatives **30** and **31** have the most favorable pharmacokinetic properties.

In summary, in silico analysis predicted drug-like properties, high GI absorption, and BBB penetration for compounds **25**, **26** (with the lowest MIC), **30**, **31**, and **32** (with intermediate MIC). In addition, compounds **30** and **31** showed excellent pharmacokinetic properties, including not being a P-gp substrate and not being CYP2C9, CYP2D6, or CYP3A4 inhibitors. These predictions would help us to design future benzo[*b*]thiophene derivatives. 

## 3. Materials and Methods

A Bruker spectrometer operating at 400 and 100 MHz was used to record ^1^H and ^13^C NMR spectra, respectively. Electron ionization (EI) and direct probe sample introduction were used in a VG-70S magnetic sector mass spectrometer for recording high-resolution mass spectra (HRMS). Thin-layer chromatography was performed using glass plates coated with silica gel 60 F_254,_ and short-wave UV light was used to visualize the molecules to monitor the progress of reactions. ACS-grade hexanes and ethyl acetate were used as the eluent for flash chromatography, and silica gel (60–120 mesh) was used as the stationary phase. Benzo[*b*]thiophenes **10**–**27** were synthesized according to procedures in the literature, and the characterization data were in good agreement with previously reported data [48,49].

### 3.1. General Procedure for the Electrophilic Cyclization Reaction

In a 6-dram vial equipped with a magnetic stir bar, 2-alkynylthioanisole (0.3 mmol) and CuSO_4_·5H_2_O (1.5 mmol) were added, followed by 5 mL of EtOH. Finally, the desired sodium halide (1.5 mmol) was added to the reaction mixture in one portion, with continued stirring overnight. The reaction mixture was filtered using celite and concentrated under vacuum. The resulting concentrated reaction mixture was absorbed on silica gel, and the final product was purified by column chromatography using hexanes and ethyl acetate as the eluent.

#### 3.1.1. 3-chloro-2-cyclohexylbenzo[*b*]thiophene (28)

Product was isolated as a pale yellow oil: ^1^H NMR (400 MHz, chloroform-*d*) δ1.25–1.40 (m, 1H), 1.40–1.58 (m, 4H), 1.75–1.85 (m, 1H), 1.85–195 (m, 2H), 2.00–2.13 (m, 2H), 3.18–3.30 (m, 1H), 7.34 (td, *J =* 1.2, 7.6 Hz, 1 H), 7.42 (td, *J =* 0.8, 7.6 Hz, 1 H), 7.50–7.80 (m, 2H); ^13^C NMR (100 MHz, chloroform-*d*) δ 26.0, 26.7, 34.1, 38.5, 115.7, 121.4, 122.7, 124.7, 124.9, 136.3, 137.3, 145.3 HRMS (EI^+^, *m*/*z*) calcd for (C_14_H_15_ClS)^+^ 250.0583, found 250.0585.

#### 3.1.2. 3-bromo-2-cyclohexylbenzo[*b*]thiophene (29)

Product was isolated as a pale yellow oil: ^1^H NMR (400 MHz, chloroform-*d*) δ1.30–1.40 (m, 1H), 1.40–1.60 (m, 4H), 1.75–1.85 (m, 1H), 1.85–1.98 (m, 2H), 2.05–2.18 (m, 2H), 3.19–3.33 (m, 1H), 7.34 (td, *J =* 1.2, 8.0 Hz, 1 H), 7.43 (td, *J =* 0.8, 7.2 Hz, 1 H), 7.76–7.81 (m, 2H); ^13^C NMR (100 MHz, chloroform-*d*) δ 26.1, 26.8, 34.3, 40.2, 104.2, 122.7, 122.8, 124.8, 125.1, 136.9, 138.6, 147.2; HRMS (EI^+^, *m*/*z*) calcd for (C_14_H_15_BrS)^+^ 294.0078, found 248.0082.

#### 3.1.3. 2-(3-chlorobenzo[*b*]thiophen-2-yl)propan-2-ol (30)

Product was isolated as an off-white solid: mp 99–101 °C; ^1^H NMR (400 MHz, chloroform-*d*) δ 1.81 (s, 6H), 2.36 (s, 1H), 7.36 (td, *J =* 1.2, 8.0 Hz, 1H), 7.43 (t, *J =* 8.0 Hz, 1H), 7.77 (d, *J =* 8.0 Hz, 2H); ^13^C NMR (100 MHz, chloroform-*d*) δ 29.9, 72.9, 113.9, 121.7, 122.7, 125.1, 125.3, 135.7, 138.6, 147.1; HRMS (EI^+^, *m*/*z*) calcd for (C_11_H_11_ClOS)^+^ 226.0219, found 226.0214.

#### 3.1.4. 2-(3-bromobenzo[*b*]thiophen-2-yl)propan-2-ol (31)

Product was isolated as an off-white solid: mp 92–94 °C; ^1^H NMR (400 MHz, chloroform-*d*) δ 1.83 (s, 6H), 2.22 (bs, 1H), 7.36 (td, *J =* 1.2, 7.2 Hz, 1H), 7.43 (dt, *J =* 0.8, 7.2 Hz, 1H), 7.78 (m, 2H); ^13^C NMR (100 MHz, chloroform-*d*) δ 29.9, 73.2, 101.5, 122.5, 123.0, 125.3, 125.4, 136.3, 140.1, 148.9; HRMS (EI^+^, *m*/*z*) calcd for (C_11_H_11_BrOS)^+^ 269.9714, found 269.9726.

#### 3.1.5. 1-(3-chlorobenzo[*b*]thiophen-2-yl)cyclopentan-1-ol (32)

Product was isolated as a pale yellow oil; ^1^H NMR (400 MHz, chloroform-*d*) δ 1.85–2.08 (m, 4H), 2.08–2.19 (m, 2H), 2.31–2.50 (m, 3H), 7.36 (t, *J =* 7.2 Hz, 1H), 7.43 (t, *J =* 7.6 Hz, 1H), 7.77 (d, *J =* 8.0 Hz, 1H), 7.78 (d, *J =* 8.0 Hz, 1H); ^13^C NMR (100 MHz, chloroform-*d*) δ 24.3, 41.1, 82.3, 114.9, 121.6, 122.6, 125.2, 125.3, 135.9, 138.5, 144.9.

### 3.2. Chemicals and Microbial Strains

The stock solutions of the compounds to be tested for antimicrobial activity were prepared in dimethyl sulfoxide (DMSO) as solvent and stored at –20 °C. The antimicrobial activity was tested against a total of six bacterial strains (three Gram-positive and three Gram-negative bacteria) and one fungal strain, namely *Bacillus cereus* (ATCC 10876), *Enterococcus faecalis* (ATCC 29212), *Staphylococcus aureus* (ATCC 29213), *Escherichia coli* (ATCC 25922), *Klebsiella pneumoniae* (ATCC 700603), *Pseudomonas aeruginosa* (ATCC 27853), and *Candida albicans* (ATCC 90028). All bacterial and fungal strains were purchased from American Type Culture Collection (ATCC). The cells were maintained according to the recommendation of CLSI in tryptic soy agar (TSA, BD Bacto™ DF0370173) for bacteria and potato dextrose agar (PDA, BD Difco DF0013176) plates for fungi [51,52].

### 3.3. Determination of Minimum Inhibitory Concentration Values

The broth micro-dilution method was used to determine the minimum inhibitory concentration (MIC) values in accordance with the Clinical and Laboratory Standards Institute (CLSI) guidelines [51,52]. In summary, the test compounds were serially twofold diluted into 96-well microplates with cation-adjusted Muller Hinton Broth (CAMHB, BD BBL™ Cat number B12322) for bacteria and RPMI-1640 (Bioworld 306110981) for the fungus to achieve the final range of test concentrations of −512–8 μg/mL. The inoculum suspension was prepared by the colony-pick method, and the turbidity was adjusted to 0.5 McFarland standard. This suspension was further adjusted to obtain the final inoculum of 5 × 10^5^ CFU/mL per well for bacteria and 2.5 × 10^3^ CFU/mL per well for fungus. The 96-well plates were sealed and incubated for 16–18 h at 35 °C for bacteria and 24–48 h at 35 °C for fungus. Once the plates were retrieved from the incubator, the absorbance was read at 600 nm wavelength using the PerkinElmer instrument program. The MIC was determined as the lowest concentration of test compound able to inhibit the visible growth of bacteria. The concentration range tested for the 3-halobenzo[*b*]thiophenes was 512 µg/mL to 8 µg/mL. All tests were performed in triplicate, and the highest MIC value obtained was reported. Four controls comprising medium with standard antibiotic (positive control), medium with DMSO (solvent control), medium with inoculum bacterial cells (negative control), and medium with broth only (negative growth control) were included in each test. Ampicillin, chloramphenicol, and kanamycin were the standard antibiotics used for antibacterial studies, while fluconazole was used as a standard for the antifungal studies.

### 3.4. Time-Kill Assay

Time-kill assays were performed using the broth macro-dilution method in accordance with the CLSI manual M-26A [57]. The experiments were performed in triplicate. Inoculum suspensions with approximately 5 × 10^5^ CFU/mL of exponentially growing bacterial cells were used in this study. The test compound was two-fold serially diluted in borosilicate glass test tubes using CAMHB with final concentrations corresponding to 0.5× MIC, MIC, and 2× MIC value. A growth control comprising the bacterial strain without the test compound was included in each trial. 

The inoculum cultures were incubated at 35 °C, and 100 µL aliquots were removed from the test tubes after timed intervals of incubation (i.e., 0, 1, 2, 4, 8, 12, and 24 h). The aliquots were serial tenfold diluted in saline as needed and plated on tryptic soy agar (TSA) plates. All plates were incubated at 35 °C for 24 h. The numbers of viable cells were determined by the plate count technique. Data were analyzed by plotting the log_10_ colony forming unit per milliliter (CFU/mL) versus time (hours). In the time-kill curve, the change in bacterial concentration is analyzed over time. The bactericidal activity of a compound is defined as the reduction of viable bacterial cell count ≥3log_10_ CFU/mL as compared to the initial inoculum, while bacteriostatic activity corresponds to <3 log_10_ CFU/mL decrease in viable bacterial cell count relative to the initial inoculum.

### 3.5. Predicted In Silico ADME Properties

Swiss ADME software (https://www.swissadme.ch, accessed on 9 November 2021) was used to predict the physicochemical and pharmacokinetic properties of all 23 compounds in the study; these properties are vital determinants to predict a good drug/lead-like molecule [59].

## 4. Conclusions

In this study, we concluded that the bromo- and chloro-substituted cyclohexanol benzo[*b*]thiophene derivatives (**25** and **26**) showed the lowest MIC activity against Gram-positive bacteria (*S. aureus*, *E. faecalis*, and *B. cereus*) and *C. albicans*. In addition, compound **25** showed rapid bactericidal activity against *S. aureus* at MIC. Using in silico methods, both **25** and **26** compounds were found to exhibit excellent drug-like properties, high GI absorption, and BBB penetration. Our data suggest that compound **25** could be a potent antibacterial and antifungal candidate, deserving of further investigation and mechanistic studies. In addition, the alcohol substitution seemed to enhance, whereas the iodo-substitution seemed to decrease, the antimicrobial activity of the benzo[*b*]thiophene derivatives of the compounds. The synthesized simple novel compounds possessed interesting attributes that could justify further confirmatory reactions to increase the number of new derivatives in future studies.

## Figures and Tables

**Table 1:** MIC value against Gram-positive bacteria and fungi. The concentration is provided in µg/mL. The maximum concentration tested was 512 µg/mL. If the compound showed no/less inhibition until 512 µg/mL, the MIC is referred to as > 512 µg/mL.

Compound		Bacteria		Fungi
	*S. aureus*	*E. faecalis*	*B. cereus*	*C. albicans*
**10**	>512	>512	>512	>512
**11**	>512	>512	>512	>512
**12**	>512	>512	>512	>512
**13**	>512	>512	>512	>512
**14**	>512	>512	>512	512
**15**	>512	>512	>512	512
**16**	>512	>512	>512	>512
**17**	>512	>512	>512	>512
**18**	>512	>512	>512	>512
**19**	**256**	**256**	**128**	**128**
**20**	>512	>512	>512	>512
**21**	>512	>512	>512	>512
**22**	512	>512	512	512
**23**	512	>512	512	256
**24**	>512	>512	>512	>512
**25**	**16**	**16**	**16**	**16**
**26**	**16**	**16**	**16**	**32**
**27**	>512	>512	>512	>512
**28**	>512	>512	>512	512
**29**	>512	>512	>512	>512
**30**	**64**	**64**	**16**	**64**
**31**	**64**	**128**	**32**	**64**
**32**	**128**	**128**	**64**	**128**
Ampicillin	8	8	32	-
Chloramphenicol	8	4	2	-
Kanamycin	2	32	2	-
Fluconazole	-	-	-	0.5

**Table 2:** In silico calculated physicochemical parameter values for benzo[*b*]thiophene derivatives using Swiss ADME software.

Compound	MW ^a^	nHA ^b^	nAHA ^c^	nRotB ^d^	nHBA ^e^	nHBD ^f^	MR ^g^	TPSA ^h^	MLOGP ^i^	ESOL ^j^
**25**	266.8	17	9	1	1	1	74.77	48.47	3.82	MS
**26**	311.2	17	9	1	1	1	77.46	48.47	3.95	MS
**30**	226.7	14	9	1	1	1	62.46	48.47	3.03	S
**31**	271.2	14	9	1	1	1	65.15	48.47	3.17	MS
**32**	252.8	16	9	1	1	1	69.96	48.47	3.57	MS

^a^ Molecular weight (MW), ^b^ number of heavy atoms (nHA), ^c^ number of aromatic heavy atoms (nAHA), ^d^ number of rotatable bonds (nRotB), ^e^ number of hydrogen bond acceptors (nHBA), ^f^ number of hydrogen bond donors (nHBD), ^g^ molecular refractivity (MR), ^h^ topological polar surface area (TPSA), ^i^ octanol/water partition coefficient (MLOGP), and ^j^ ESOL (estimated SOLubility) with MS moderately soluble and S representing soluble.

**Table 3:** In silico, calculated drug-likeness values for benzo[*b*]thiophene derivatives using Swiss ADME software.

Compound	Lipinski	Ghose	Veber	Egan	Muegge	PAINS ^a^	Brenk
**25**	Yes	Yes	Yes	Yes	Yes	0	0
**26**	Yes	Yes	Yes	Yes	Yes	0	0
**30**	Yes	Yes	Yes	Yes	Yes	0	0
**31**	Yes	Yes	Yes	Yes	Yes	0	0
**32**	Yes	Yes	Yes	Yes	Yes	0	0

^a^ PAINS (for pan assay interference structures).

**Table 4:** In silico pharmacological properties of benzo[*b*]thiophene derivatives predicted using Swiss ADME software.

Compound	GI ^a^ Absorption	BBB ^b^ Permeant	P-gp ^c^ Substrate	CYP1A2 Inhibitor	CYP2C19 Inhibitor	CYP2C9 Inhibitor	CYP2D6 Inhibitor	CYP3A4 Inhibitor	Log Kp (cm/s)
**25**	High	Yes	Yes	Yes	Yes	Yes	Yes	No	−4.86
**26**	High	Yes	Yes	Yes	Yes	Yes	Yes	No	−5.09
**30**	High	Yes	No	Yes	Yes	No	No	No	−5.33
**31**	High	Yes	No	Yes	Yes	No	No	No	−5.56
**32**	High	Yes	Yes	Yes	Yes	No	Yes	No	−5.16

**^a^** GI (gastrointestinal), **^b^** BBB (blood–brain barrier), **^c^** P-gp (permeability glycoprotein).

## Data Availability

Data are contained within the article and Supplementary Materials.

## Supplementary Materials

The following are available online at https://www.mdpi.com/article/10.3390/ph15010039/s1. Figure S1. ^1^H NMR spectra of **25**; Figure S2. ^13^C NMR spectra of **25**; Figure S3. ^1^H NMR spectra of **26**; Figure S4. ^13^C NMR spectra of **26**; Figure S5. ^1^H NMR spectra of **28**; Figure S6. ^13^C NMR spectra of **28**; Figure S7. ^1^H NMR spectra of **29**; Figure S8. ^13^C NMR spectra of **29**; Figure S9. ^1^H NMR spectra of **30**; Figure S10. ^1^^3^C NMR spectra of **30**; Figure S11. ^1^H NMR spectra of **31**; Figure S12. ^13^C NMR spectra of **31**; Figure S13. ^1^H NMR spectra of **32**; Figure S14. ^13^C NMR spectra of **32**.
