# Supplementary material for: Synthesis and Evaluation of 3-Halobenzo[b]thiophenes as Potential Antibacterial and Antifungal Agents
Source: Pharmaceuticals (Basel). 2021 Dec 28;15(1):39. doi: 10.3390/ph15010039 (PMC8780876; doi:10.3390/ph15010039)
Supplement: Supplementary file 1 [file pharmaceuticals-15-00039-s001.zip › pharmaceuticals-1509706-supplementary.pdf]

*Supplementary Materials*

# Synthesis and evaluation of 3-halobenzo[*b*]thiophenes as potential antibacterial and antifungal agents

**Prerna J. Masih[1,*], Tanay Kesharwani [2], Elivet Rodriguez [1], Mia A. Vertudez [1], Mina L. Motakhaveri [1], Terelan K. Le [1], Minh Kieu T. Tran [1], Matthew R. Cloyd [2], Cory T. Kornman [2] and Aimee M. Phillips [1]**

[1] Department of Biology, University of West Florida, Pensacola, FL, 32514, USA
[2] Department of Chemistry, University of West Florida, Pensacola, FL, 32514, USA
* Correspondance: pmasih@uwf.edu

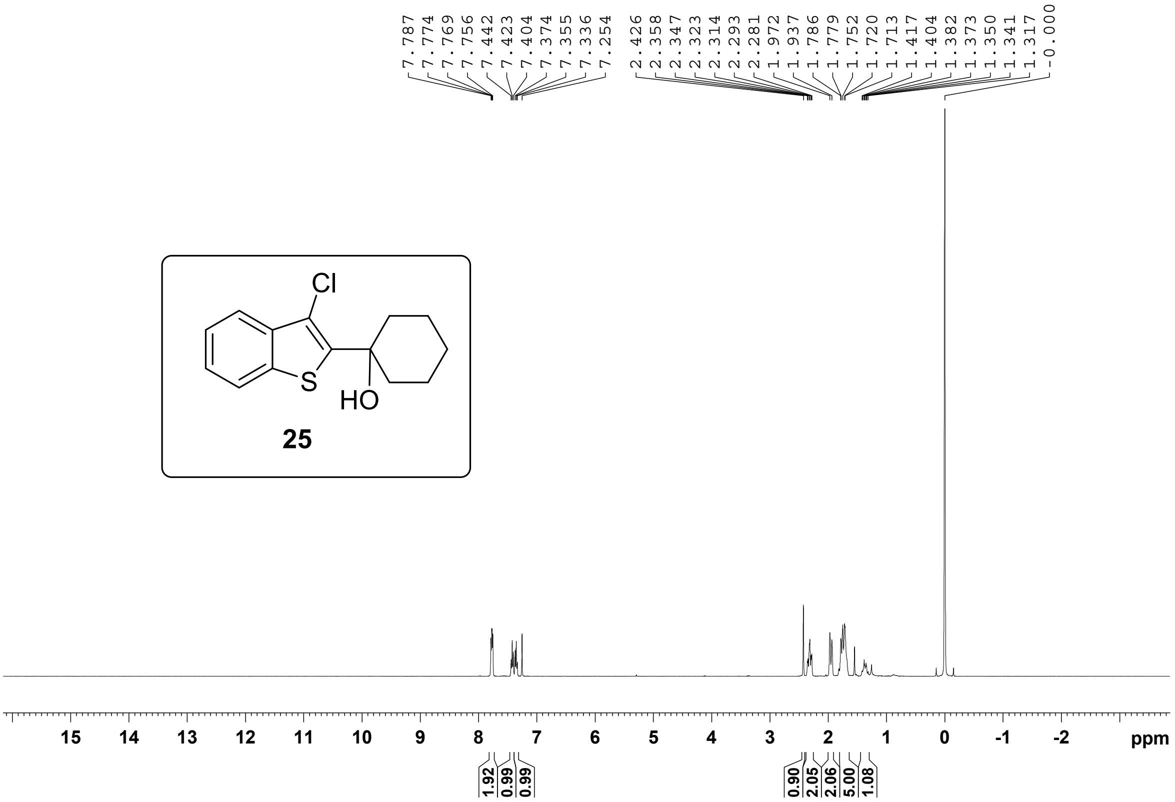


**Figure S1.** $^1$H NMR spectra of **25.**

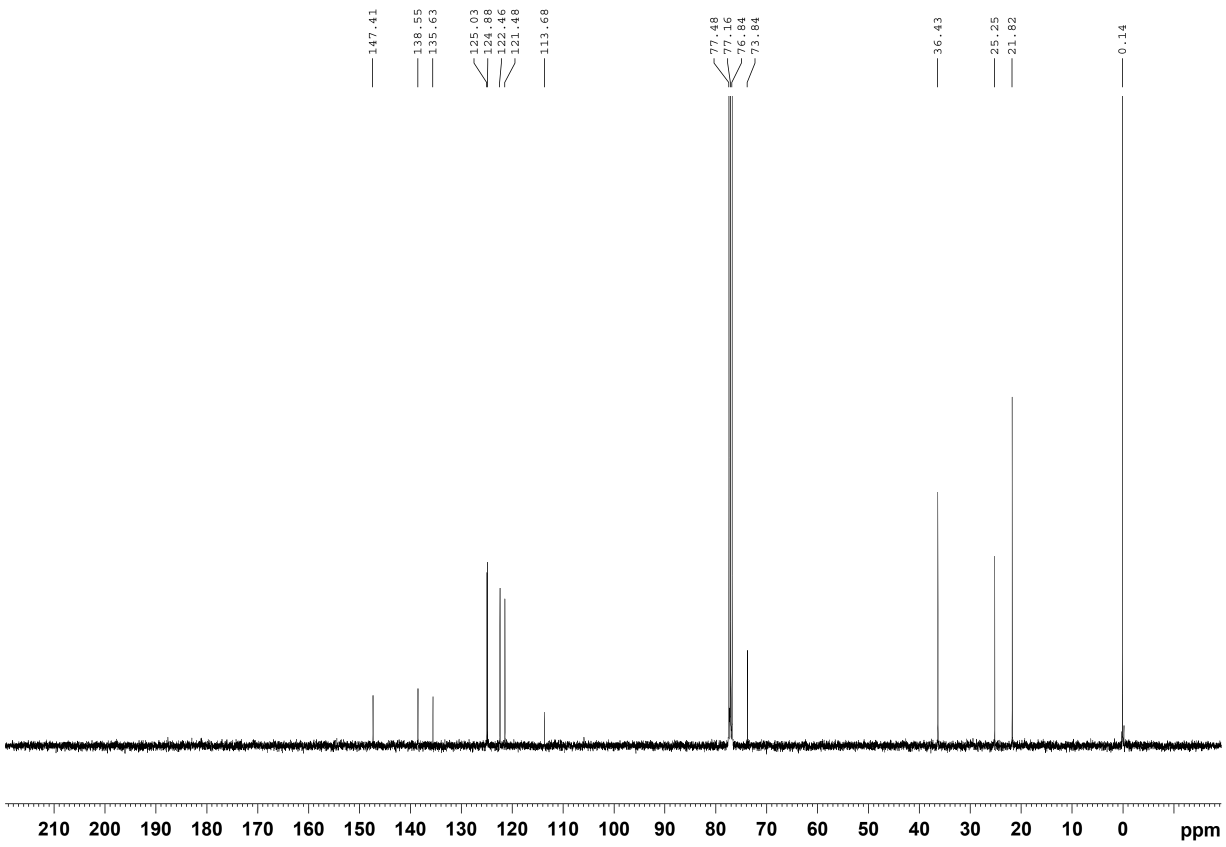


**Figure S2.** $^{13}C$ NMR spectra of **25.**

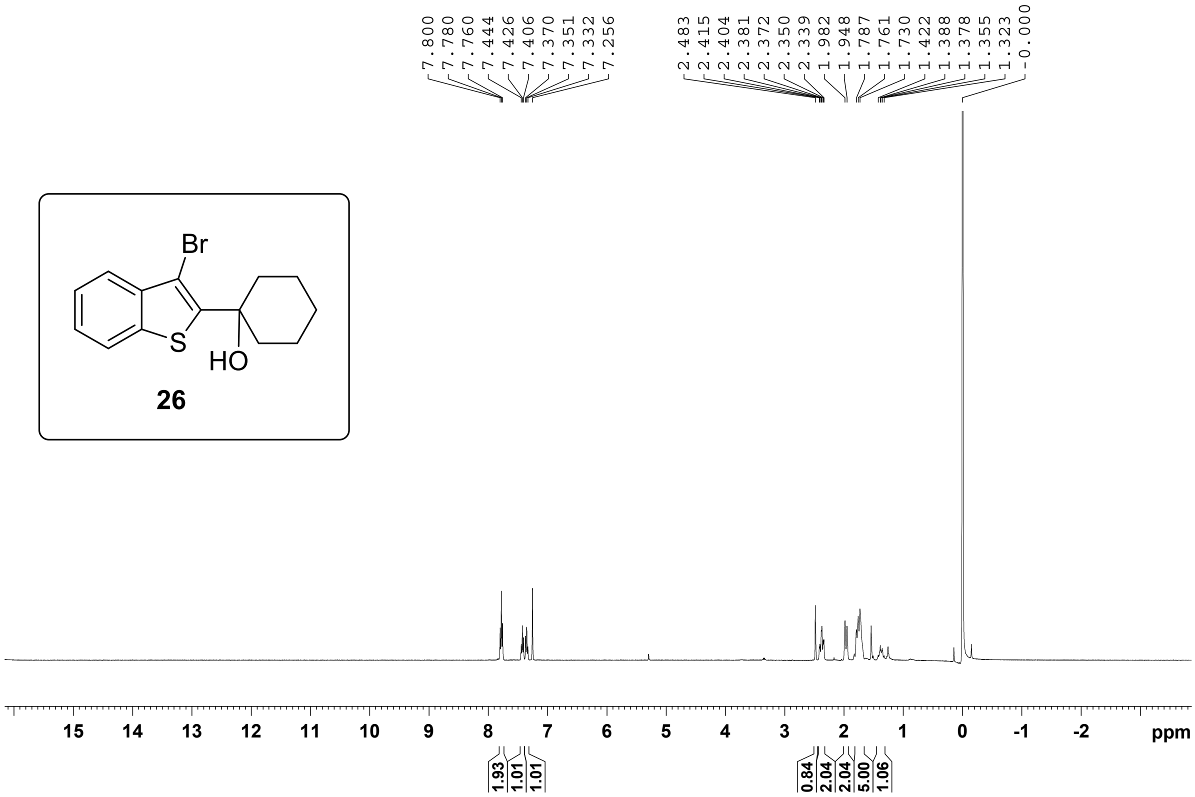


**Figure S3.** $^{1}$H NMR spectra of **26.**

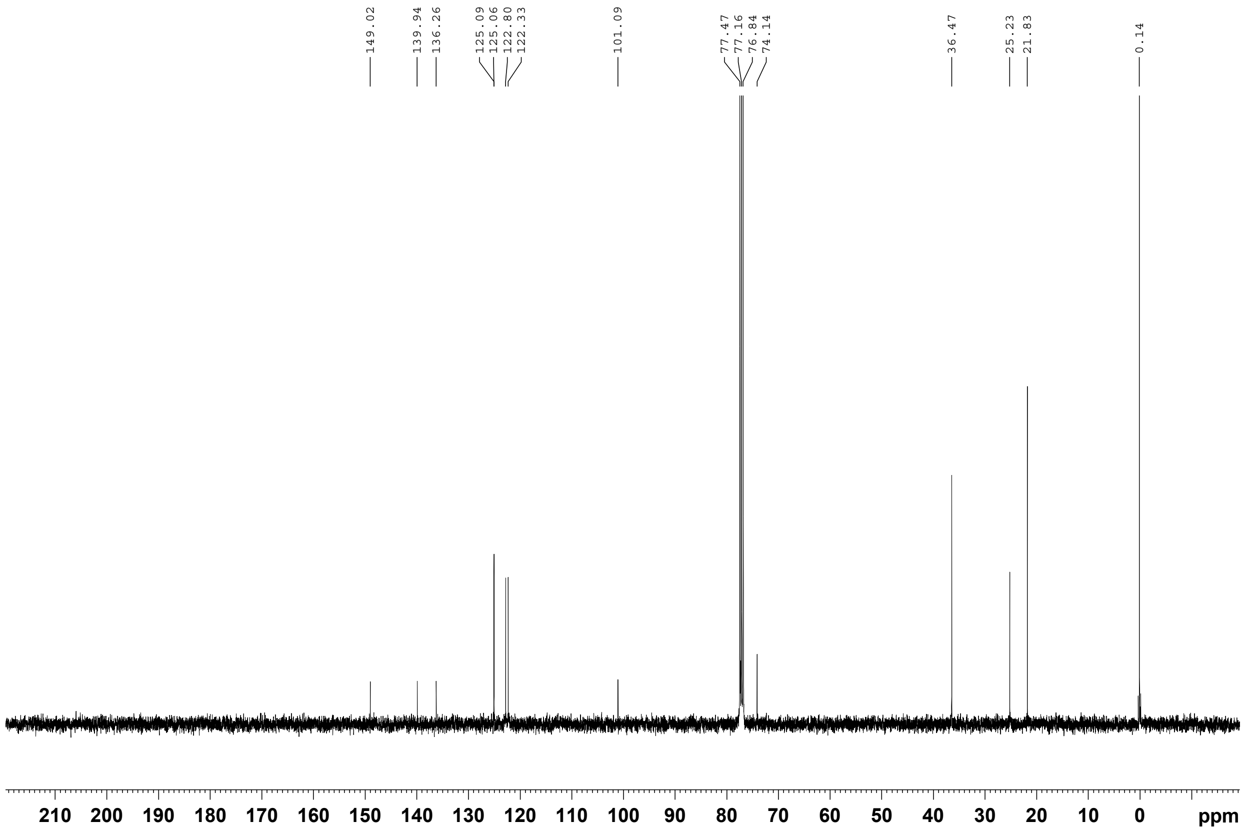


**Figure S4.** $^{13}$C NMR spectra of **26.**

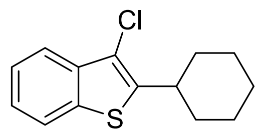


**28**

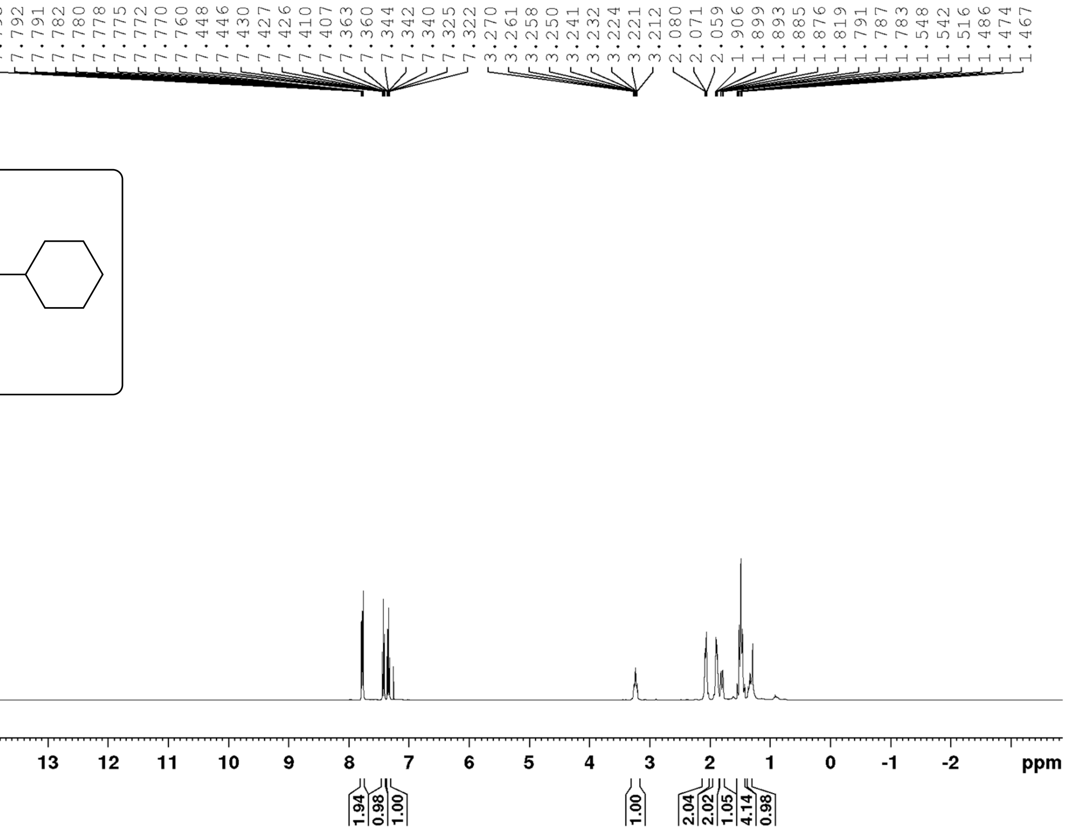


**Figure S5.** $^{1}H$ NMR spectra of **28.**

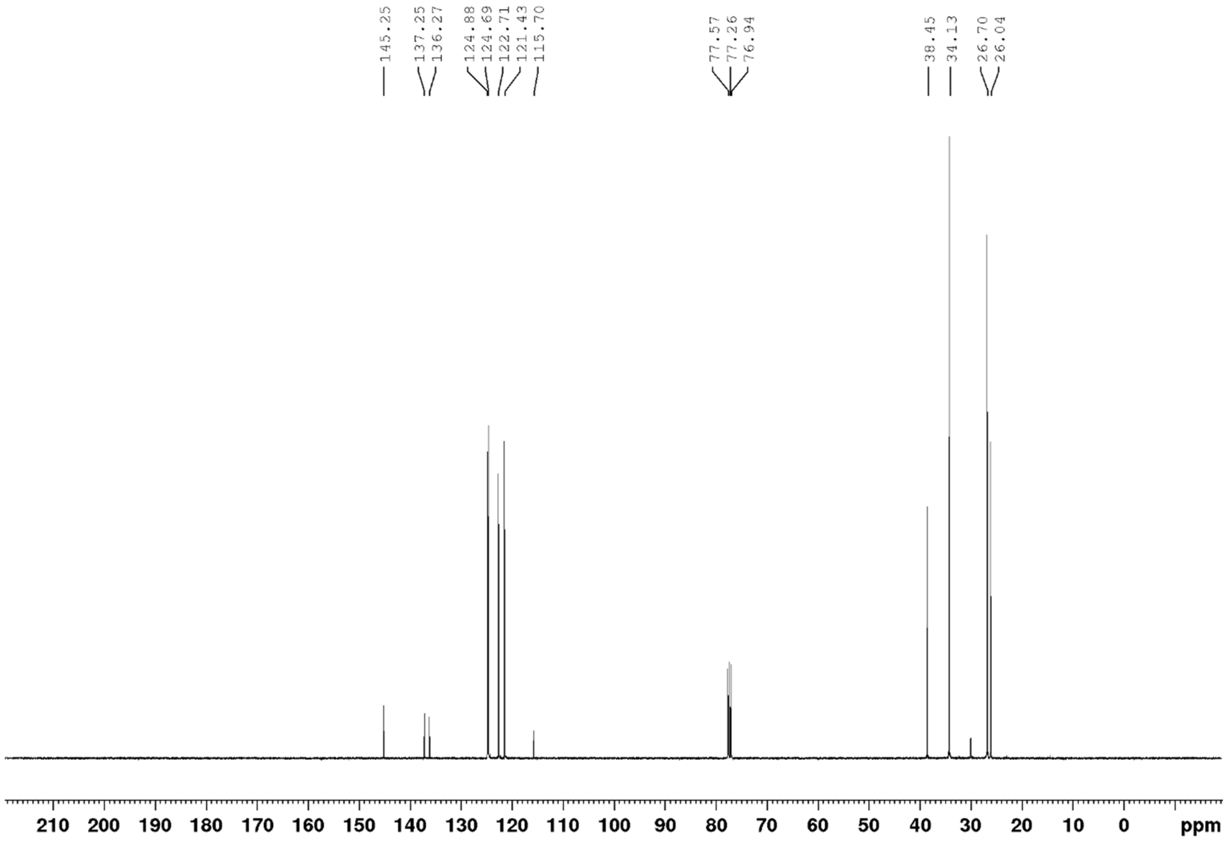


**Figure S6.** $^{13}C$ NMR spectra of **28.**

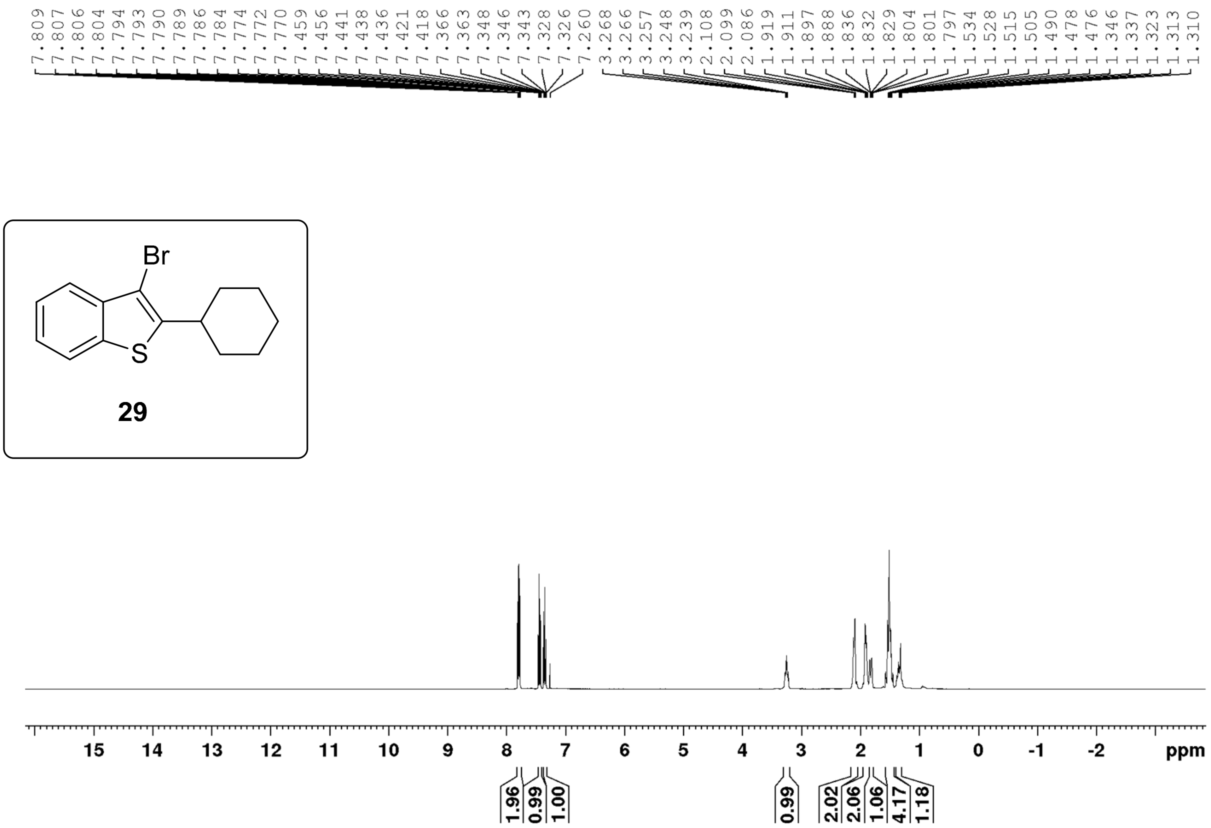


**Figure S7.** $^{1}H$ NMR spectra of **29.**

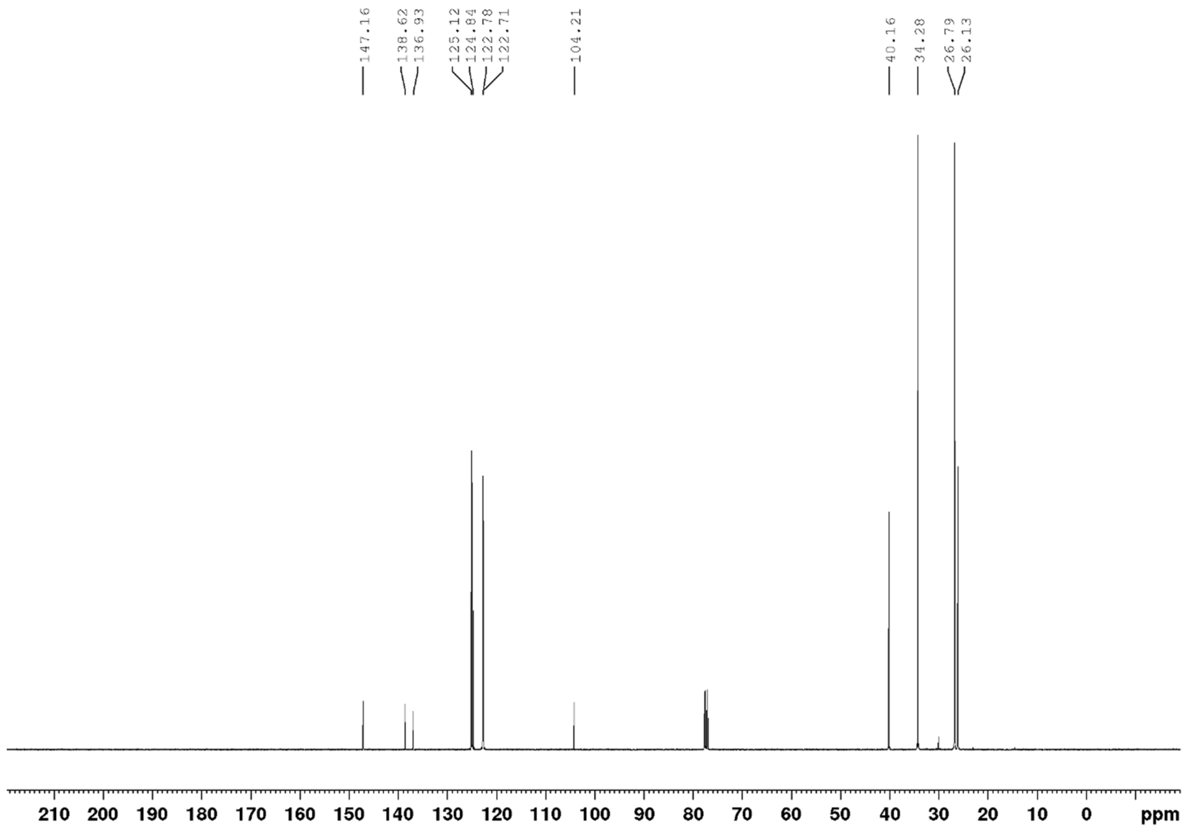


**Figure S8.** $^{13}C$ NMR spectra of **29.**

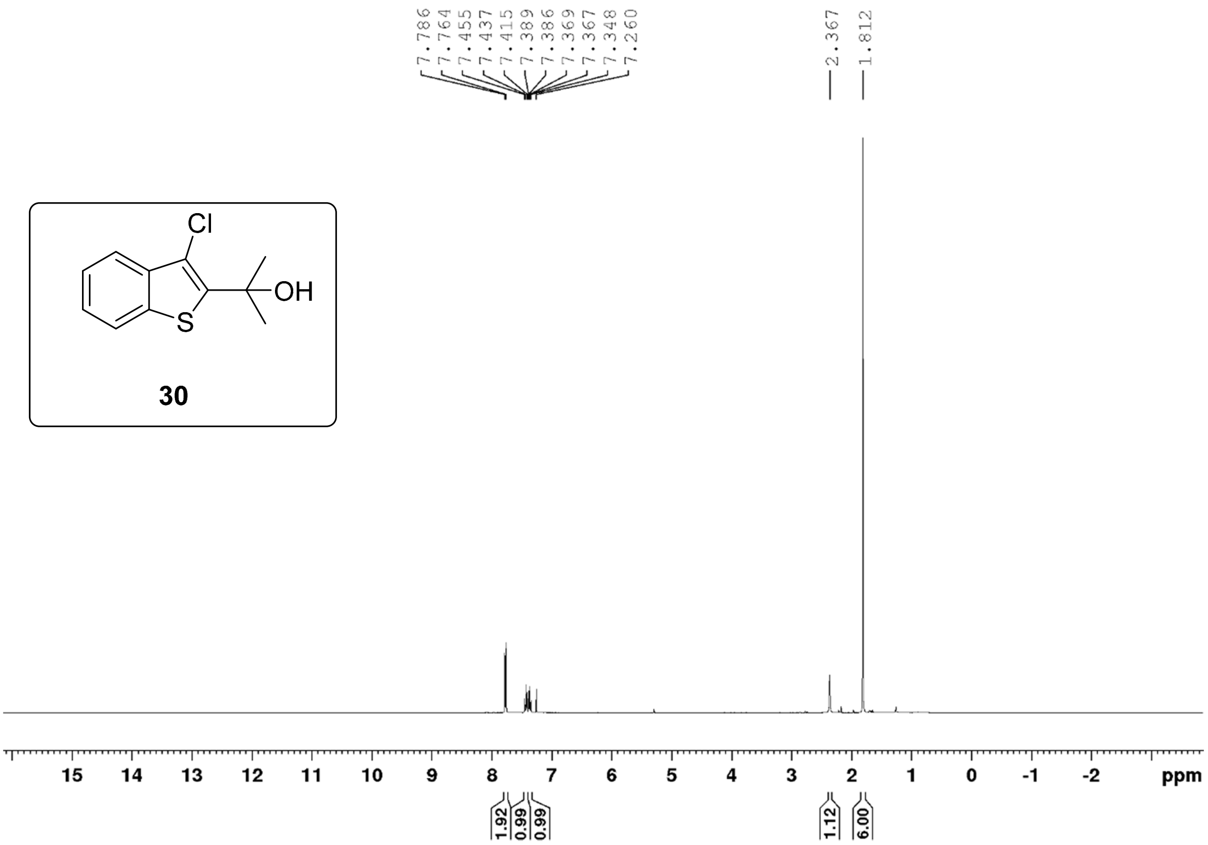


**Figure S9.** $^{1}H$ NMR spectra of **30.**

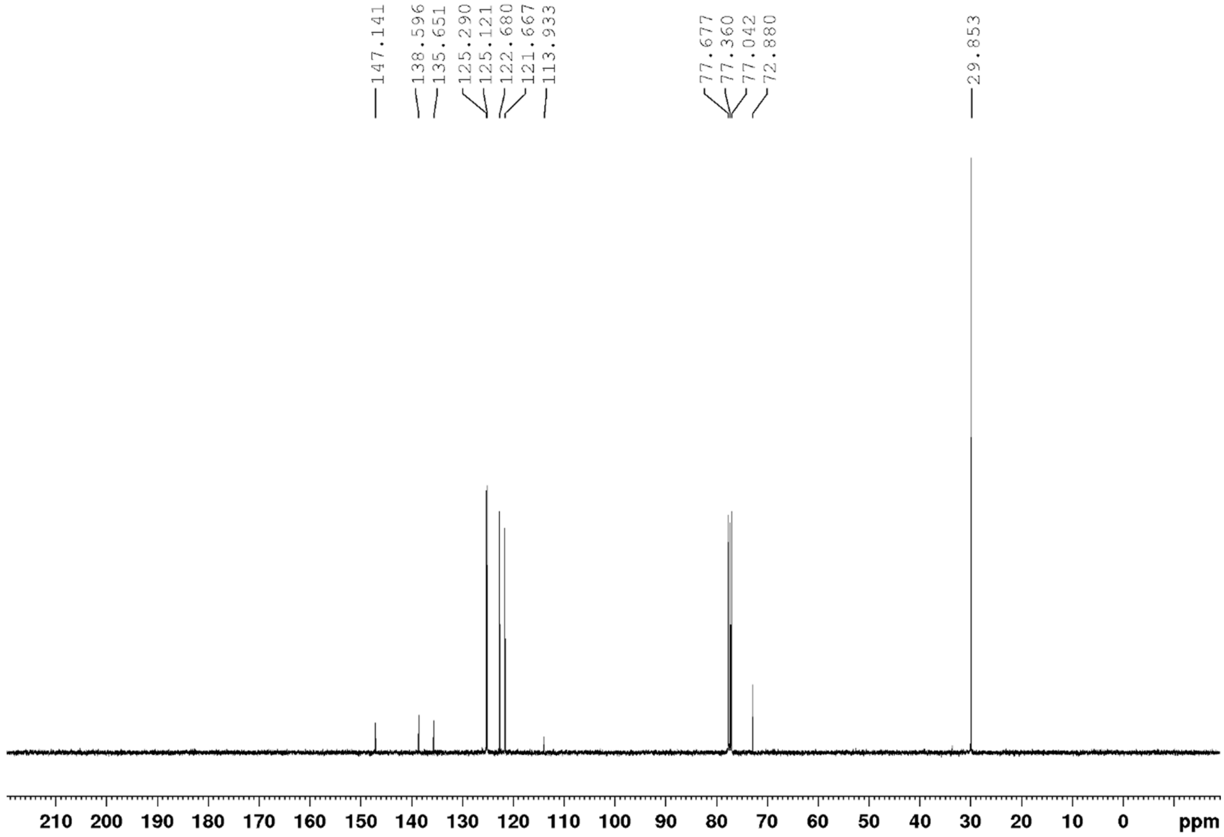


**Figure S10.** $^{13}C$ NMR spectra of **30.**

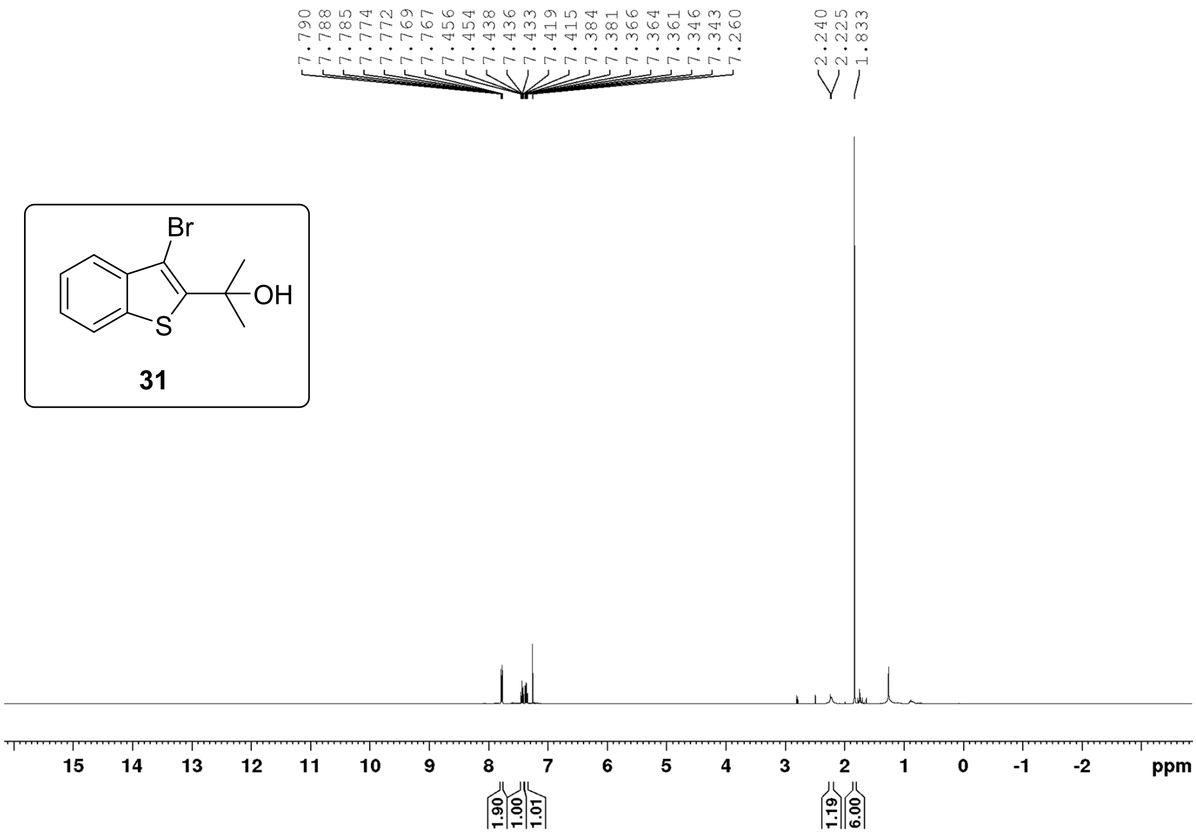


**Figure S11.** $^{1}$H NMR spectra of **31.**

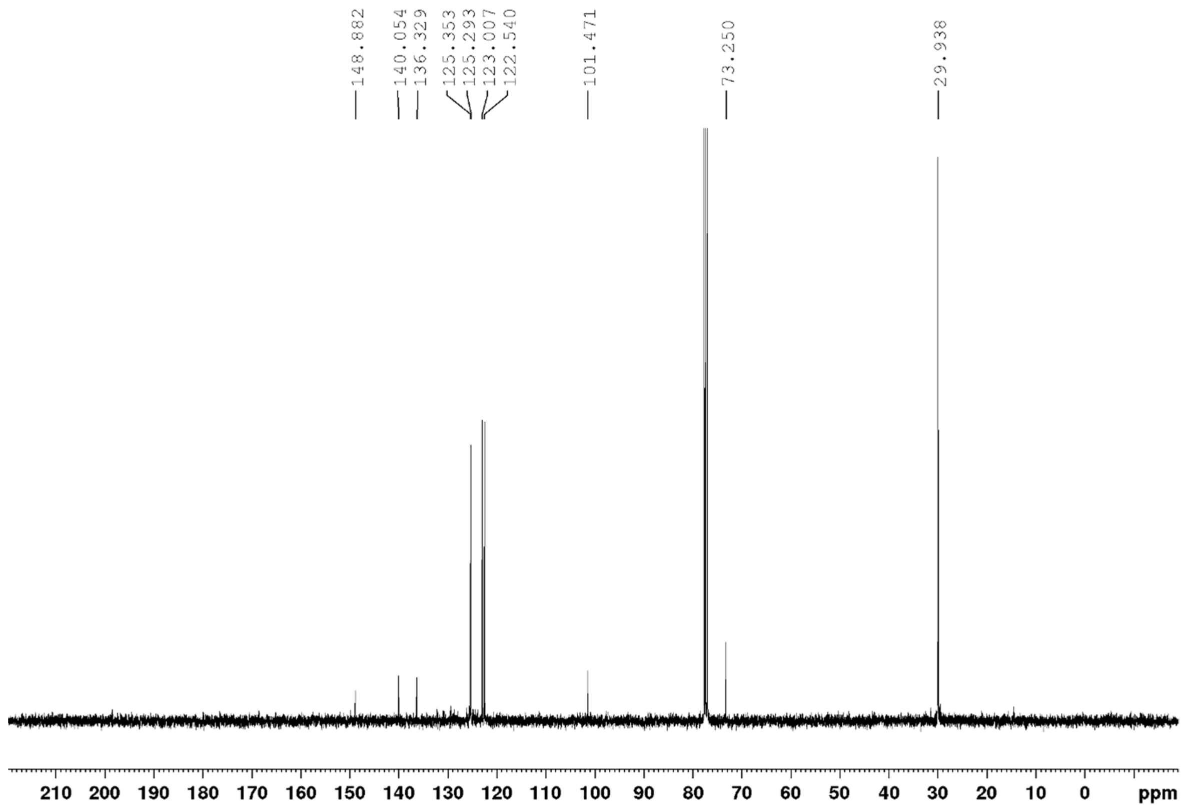


**Figure S12.** $^{13}$C NMR spectra of **31.**

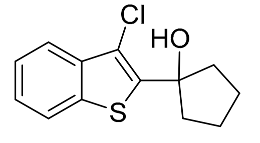


**32**

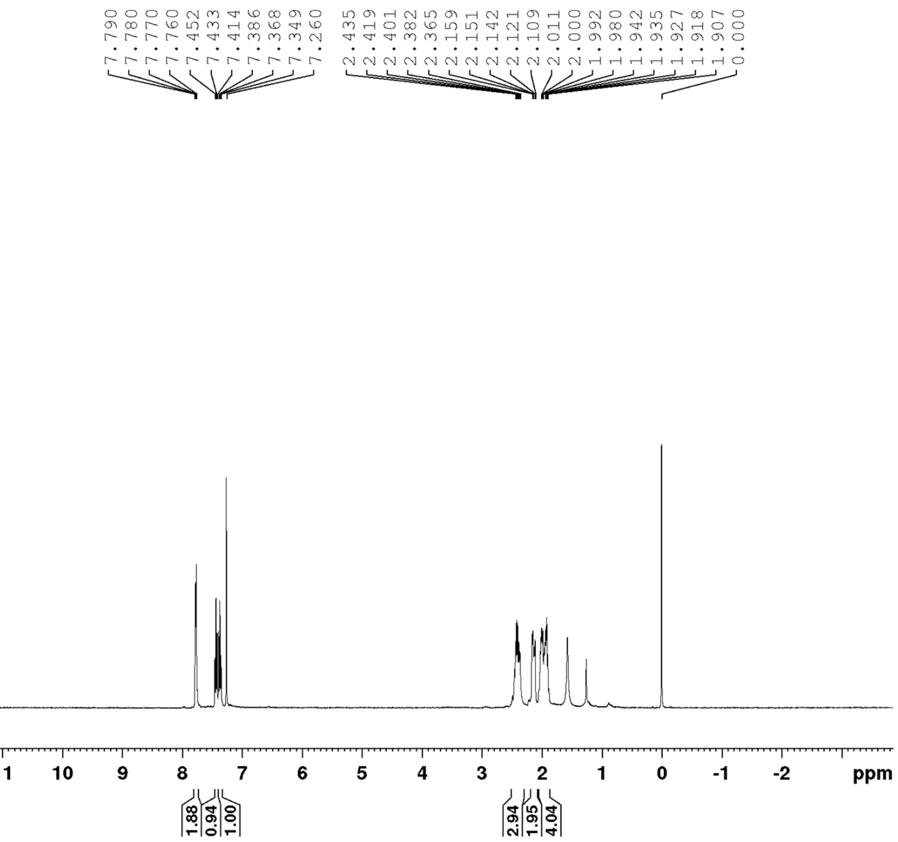


**Figure S13.** $^{1}$H NMR spectra of **32.**

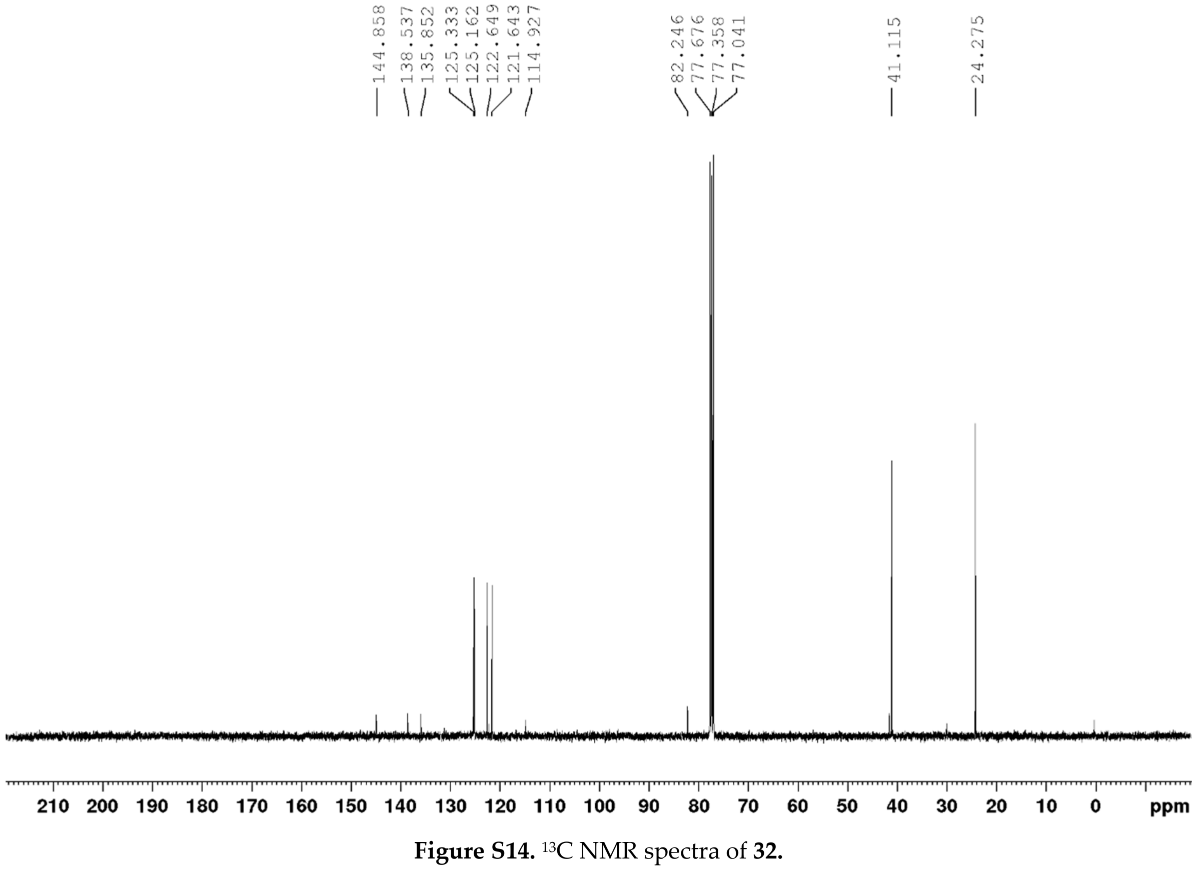


**Figure S14.** $^{13}$C NMR spectra of **32.**
